# Retraction: Atorvastatin May Attenuate Recurrence of Chronic Subdural Hematoma

**DOI:** 10.3389/fnins.2016.00465

**Published:** 2016-10-07

**Authors:** Frontiers Editorial Office

The Journal and Authors retract the 28 June 2016 article cited above for the following reasons provided by the Authors:

In one of our collaborating centers (The People's Hospital of AnQiu City), 38 patients were enrolled in an atorvastatin group. Among them, 26 patients lost to follow-up were categorized and analyzed as “non-recurrence group.” It was a fundamental error and thus we must retract this article. We apologize for any inconvenience caused by the retraction of this article.

